# When environmental changes do not cause geographic separation of fauna: differential responses of Baikalian invertebrates

**DOI:** 10.1186/1471-2148-10-320

**Published:** 2010-10-23

**Authors:** Varvara Fazalova, Bruno Nevado, Tatiana Peretolchina, Jeanna Petunina, Dmitry Sherbakov

**Affiliations:** 1 Laboratory of Molecular Systematics, Limnological Institute of the Siberian Branch of the Russian Academy of Sciences, Ulan-Batorskaya 3, 664033 Irkutsk, Russia; 2 Evolution and Ecology Program, International Institute for Applied Systems Analysis, Schlossplatz 1, A-2361 Laxenburg, Austria; 3 Vertebrate Department, Royal Belgian Institute of Natural Sciences, Vautierstraat 29, 1000 Brussels, Belgium; 4 Evolutionary Ecology Group, University of Antwerp, Groenenborgerlaan 171, B-2020 Antwerp, Belgium; 5 Faculty of Biology and Soil Science, Irkutsk State University, Sukhe-Batora 5, 664003 Irkutsk, Russia

## Abstract

**Background:**

While the impact of climate fluctuations on the demographic histories of species caused by changes in habitat availability is well studied, populations of species from systems without geographic isolation have received comparatively little attention. Using CO1 mitochondrial sequences, we analysed phylogeographic patterns and demographic histories of populations of five species (four gastropod and one amphipod species) co-occurring in the southwestern shore of Lake Baikal, an area where environmental oscillations have not resulted in geographical isolation of habitats.

**Results:**

Species with stronger habitat preferences (gastropods *B. turriformis*, *B. carinata *and *B. carinatocostata*) exhibit rather stable population sizes through their evolutionary history, and their phylogeographic pattern indicates moderate habitat fragmentation. Conversely, species without strong habitat preference (gastropod *M. herderiana *and amphipod *G. fasciatus*) exhibit haplotype networks with a very abundant and widespread central haplotype and a big number of singleton haplotypes, while their reconstructed demographic histories show a population expansion starting about 25-50 thousand years ago, a period marked by climate warming and increase in diatom abundance as inferred from bottom-lake sedimentary cores.

**Conclusions:**

In agreement with previous studies, we found that species reacted differently to the same environmental changes. Our results highlight the important role of dispersal ability and degree of ecological specialization in defining a species' response to environmental changes.

## Background

Many studies have demonstrated the strong influence of climate fluctuations on the patterns of genetic diversity of species. Continental glaciations resulted in geographic isolation of terrestrial species by affecting habitat availability [1,2]. After the climate warming, some species experienced demographic expansions and occupied newly created habitats [3-5]. Additionally, climate cooling was linked with low level of oceans and lakes [6]. When the water level decreased, marine species could experience range contractions and this again resulted in change of their phylogeographic patterns [7,8]. On the other hand, low ocean level affects the connectivity of islands and the distribution of species inhabiting them [9]. However, analysis of the demographic histories of species from northeastern Pacific showed that half of them were not affected by climatic changes in the Pleistocene [10]. This suggests that, even if the majority of studies consider geographic isolation as a driving force of changes in demographic histories, impact of climate cooling on ecological systems could be more complex. For example, switches in oceanic thermohaline circulation could change distribution and abundance of food and result in a bottleneck [11]. Furthermore, in systems where environmental changes resulted in isolation of populations, the present genetic structure of populations will reflect to a great degree the changes in genetic diversity due to random evolution in these isolated populations. As such, analysis of current patterns of diversity will be affected by this, as well as by the demographic histories of the populations, or the presence/absence of selective pressures. Conversely, in systems where geographical isolation is absent one can distinguish the effect of genetic drift in small isolated populations from the demographic changes brought about by the environmental changes themselves. It therefore seems appropriate to study ecosystems that are known to be affected by environmental changes, but where these changes did not lead to geographic separation of populations.

Ancient lakes are famous for their high level of biodiversity. Whereas many studies on speciation were devoted to the fauna of ancient lakes, reconstruction of their demographic histories received little attention (but see e.g. [12]). Lake Baikal is the largest freshwater continental ecosystem [13], and given its high-latitude location it is particularly sensitive to climatic variations [14]. Despite the great depth of the lake (c. 1650 m), its water is well oxygenated throughout, creating unique habitats. The sediments of the lake are one of the most valuable continental climatic archives having uninterrupted record back to Late/Middle Miocene [15,16]. The paleoclimatic history of Lake Baikal was reconstructed based on records of diatom and associated biogenic silica in sediments, their variation corresponding to the Marine Isotope Stages (MIS) of climate change [17-23]. Additionally, sedimentary photosynthetic pigments provide more data about past productivity of the lake by representing the whole assemblage of phytoplankton [24,25]. Numerous strong environmental changes were identified during the Upper and Middle Pleistocene [21,26-29] and the Holocene [18,30,31]. Also there is evidence for water level fluctuations during periods of climatic cooling [32,33]. While Lake Baikal is known to have been affected by environmental changes, the lake's geological structure suggests that these changes have not affected the connectivity of habitats. Previous studies on the genetic variation of invertebrates from the lake revealed variation of population dynamics presumably caused by geological events (tectonic shifts), changes in global climate and related changes in sedimentation rate [34].

Recent studies comparing the demographic histories of multiple co-occurring species [10,35,36] found that populations of these species responded in different ways to the same environmental changes. However, exact causes for such differences were often difficult to distinguish given the numerous biological differences between the investigated species. We thus compare, in this study, species with very similar biological traits and life-history characteristics, allowing us to identify the factors responsible for the species' differential response to environmental changes. This approach has already proved valuable in understanding key aspects of the response of species to fluctuation of environmental conditions (e.g. see [37,38]). We focus on four gastropod species of the family Baicaliidae: *Baicalia carinata *(W. Dybowski, 1875) is an abundant sand dwelling species with a circum-lacustrine distribution; *Baicalia carinatocostata *(W. Dybowski, 1875) is often found in sandy habitats together with *B. carinata *but usually in smaller numbers; *Maackia herderiana *dominates the rocky surfaces but is also found in lower abundance in sandy and silty substrates in the southwestern shore of the lake [39]; and *Baicalia turriformis *(W. Dybowski, 1875) inhabits rocks along the same shoreline as *M. herderiana *(Lindholm, 1909). The four species also use different egg-laying substrate: *B. carinata *lays its eggs on the surface of the shell of other conspecifics; *B. carinatocostata *lays its eggs in sand; *M. herderiana *uses cavities of stones; and *B. turriformis *attaches its eggs to smooth surfaces of rocks [40,41]. Juveniles of these gastropods emerge directly from egg capsules and therefore the dispersal ability of these species is low when compared to other gastropods with free-swimming, planktonic larvae. For comparative purposes, we included in this study data from the amphipod *Gmelinoides fasciatus *(Stebbing, 1899). It is found in high abundances in sandy and rocky bottoms in almost all littoral zones of the lake at water depths between 0 and 5 meters. This species is a successful invader that rapidly increases its population size when introduced in new ecosystems [42,43] and this suggests that it might represent a good proxy for habitat and food availability. All four gastropod species and *G. fasciatus *are suspension feeders and have similar dietary preferences. Observations on the stomach content of the gastropods showed that they mainly consume planktonic diatoms *Aulacoseira baicalensis*, *A. islandica*, *Cyctotella baicalensis *and *C. minuta *[44]. These diatom species significantly contribute to the paleo-record of the lake and their abundance reflects the bioproductivity of the ecosystem. It therefore seems appropriate to use the paleoclimatic history (based on record of sediments) to study the impact of past environmental changes on the species herein investigated.

In this study we investigated how demographic histories of several co-occuring species with different ecological preferences were affected by environmental changes in an ecosystem where these changes did not cause geographical separation of fauna. To this end we collected mitochondrial DNA data (CO1) from populations of five species from the southwestern shore of Lake Baikal. We examined phylogeographic patterns and performed comparative analysis of the demographic histories of these populations in view of the known past environmental changes.

## Results

**Figure 1:**
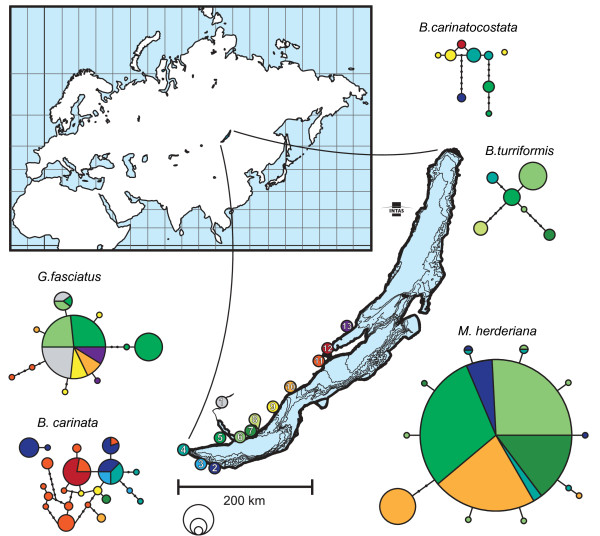
**Sampling localities and haplotype networks of the studied species**. Diameter of circles representing haplotypes in the networks are proportional to the number of sequences per haplotype (empty circles below lake scale represent sizes for 2, 5 and 10 individuals), colours represent locality of origin, empty small circles represent missing haplotypes. Throughout shoreline, coloured circles represent sampled localities, numbers inside these circles correspond to those in Table 1.

Our taxon sampling included 222 individuals from the five targeted species collected from 13 localities. The list of sampling localities is shown in Table 1 (for details please see Additional file 1). Haplotype networks for the investigated species show different patterns of genetic variation (Figure 1). Most of *M. herderiana *individuals carry the same haplotype and there is a number of singleton haplotypes, this suggests population growth. The same pattern is exhibited by *G. fasciatus*, with a dominating haplotype and a small number of less abundant haplotypes. Conversely, haplotypes found in *B. carinata*, *B. carinatocostata *and *B. turriformis *are very diverse and distributed throughout the network. In *B. carinata *and *B. carinatocostata *very different haplotypes were found in several localities, some of which were resolved in separate networks.

**Table 1:** Sampling localities and number of samples of each species used in this study

Locality	**Locality No**.	*B. carinata*	*B. carinatocostata*	*B. turriformis*	*G. fasciatus*	*M. herderiana*
Angara River	1	-	-	-	7	-

Murinskaya Banka	2	14	2	-	-	5

Utulik	3	3	-	-	-	-

Kultuk	4	5	6	3	-	4

Polovinnaya Bay	5	-	4	5	17	16

Listvyanka	6	-	-	10	7	19

Bolshie Koty	7	2	-	3	-	9

Varnachka	8	-	-	4	-	-

Peschanaya Bay	9	3	5	-	4	-

Bugul'deika	10	2	-	-	3	25

Tutaiskaya Bay	11	7	-	-	-	-

Olkhon Gates	12	21	2	-	2	-

Zunduk Cape	13	-	-	-	3	-

Total No.	-	57	19	25	43	78

Tables of pairwise F_ST _values and their significance levels for each studied species are shown in Additional file 2. The studied species exhibited variable level of geographic structuring, from total absence of significant F_ST _values between pairs of localities (*G. fasciatus*) to significant F_ST _values between almost all of these comparisons (*B. turriformis*).

**Figure 2:**
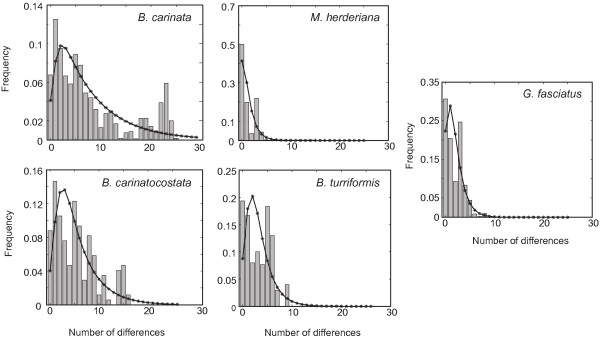
**Mismatch distributions for the studied species**. Bars represent observed values, lines represent expected values under model of sudden population growth (estimated in DNAsp).

Results of comparative analysis of mismatch distributions [45,46] for each species are depicted in Figure 2. Mismatch distributions of *M. herderiana *and *G. fasciatus *exhibit similar shape with most pairwise comparisons having small genetic distances, showing a relatively good fit to the expected mismatch distributions under the model of population growth. Conversely, the mismatch distributions of *B. carinata*, *B. carinatocostata *and *B. turriformis *are rather multimodal and ragged, and contain a higher proportion of comparisons resulting in larger genetic distances.

Table 2 summarizes the intraspecific statistics estimated for each species: number of sequences, number of segregating sites, number of haplotypes, nucleotide diversity, haplotype diversity and average number of nucleotide differences. Felsenstein [47] suggests that eight haplotypes randomly sampled from a single panmictic population allow accurate estimates of population genetics' parameters. Therefore, our sampling effort seems adequate (only for *B. turriformis *were less than 8 haplotypes recovered in this study). Despite small sample sizes, *B. turriformis *and *B. carinatocostata *exhibited high nucleotide diversity, while the lowest nucleotide diversity was found in *M. herderiana *(0.0019).

**Table 2:** Summary statistics of genetic variation for each species.

Species	N	S	h	Pi	Hd	k
*B. carinata*	57	29	21	0.0136	0.932	8.014

*B. carinatocostata*	19	24	10	0.0091	0.912	5.368

*B. turriformis*	25	12	6	0.0054	0.807	3.200

*G. fasciatus*	43	14	10	0.0033	0.693	1.834

*M. herderiana*	78	13	12	0.0019	0.501	1.123

**N **- number of sequences; **S **- number of segregating sites; **h **- number of haplotypes; **Pi **- nucleotide diversity; **Hd **- haplotype diversity; **k **- average number of nucleotide differences.

*Maackia herderiana *is the only species for which classic tests rejected the hypothesis of neutrality, with Ramos-Onsins' R2 test against population expansion being significant (see Table 3). *G. fasciatus *exhibited low, though non-significant, p-values for Ramos-Onsins R2 and Tajima's D tests. H test showed no purifying selection for any of the investigated species.

**Table 3:** Statistical tests of neutrality calculated for each species

Species	Fs	R2	D	H
*B. carinata*	-1.76(0.28)	0.137(0.86)	0.89(0.84)	0.79(0.91)

*B. carinatocostata*	-0.027(0.51)	0.122(0.41)	-0.85(0.23)	0.827(0.89)

*B. turriformis*	0.05 (0.52)	0.127(0.62)	-0.07 (0.55)	0.116 (0.31)

*G. fasciatus*	-2.64(0.164)	0.067(0.08)	-1.35(0.09)	0.684(0.81)

*M. herderiana*	-0.19 (0.56)	**0.099 (0.043)**	-0.05 (0.55)	0.05 (0.27)

**Fs **- Fu's Fs index; **R2 **- Ramos-Onsins R2 test; **D **- Tajima's D; **H **- Fay and Wu's H statistics. In parentheses, p-values are given for each statistics. Results of significant tests (p < 0.05) are shown in bold.

**Figure 3:**
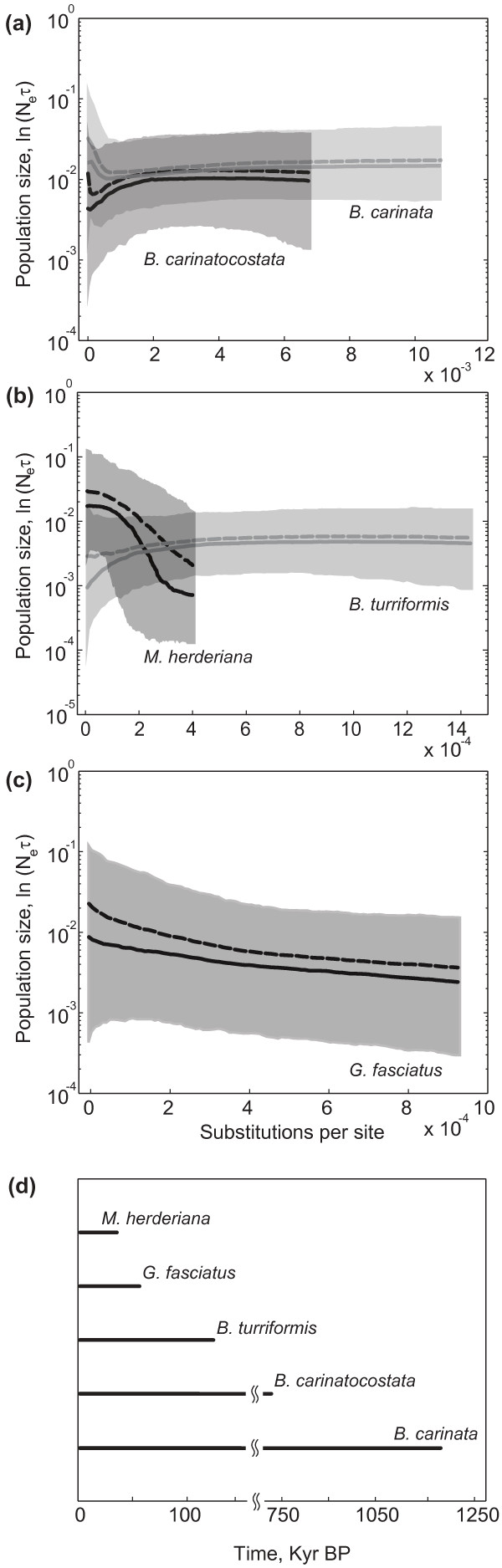
**Inferred demographic histories for sand dwellers *B. carinata *and *B. carinatocostata ***(**a), for rock dwellers *M. herderiana *and *B. turriformis *(b) and for ecologically plastic *G. fasciatus *(c).** Thick solid lines are median estimates, and the thick dashed lines are mean estimates. Grey shades show 95% highest posterior density limits. Duration of demographic histories for the five species is compared in bottom panel (d).

**Figure 4:**
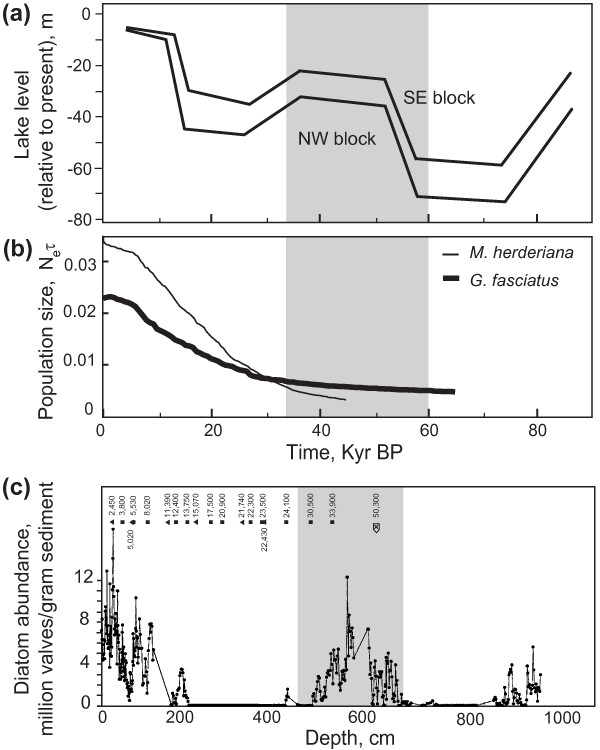
**Calibrated demographic histories of *G. fasciatus *and *M. herderiana *and reconstructed paleoclimatic history**. (a) Inferred lake water level (relative to present) based on seismic survey of the delta of the Selenga River. The two lines (NW and SE) represent results obtained using the northwestern and southeastern (respectively) blocks of the delta (see reference [32] for details); adapted from reference [32], (b) changes in population size in *G. fasciatus *and *M. herderiana *(mean estimates; obtained in this study) and (c) diatom abundance inferred from sedimentary drill core BDP-93-2 (redrawn from [21]). In (c), solid triangles are the radiocarbon dates for the core BDP-93-1 [82,83] and squares are for core BDP-93-2; the arrow indicates the 50.3 Kyr BP correlation tie point for BDP-93 record [29]. The shaded areas correspond to Marine Isotope Stage 3.

Figure 3 summarizes results of Bayesian skyline reconstructions of demographic histories for sand dwelling (a) and for rock dwelling (b) gastropod species, and also for *G. fasciatus *(c). Sand dwelling *B. carinata *and *B. carinatocostata *show rather stable population sizes as does the rock-dwelling *B. turriformis*. *Maackia herderiana *shows a dramatic population expansion and *G. fasciatus *shows signs of population growth. Results of Bayesian Skyline Plots (BSPs) were not influenced by the change of the substitution model for *G. fasciatus *(see Additional file 3). Figure 3 (d) shows the relative duration of the recovered demographic histories. The most recent common ancestors of the populations of *M. herderiana *and *G. fasciatus *are relatively recent when compared to *B. carinata*, *B. carinatocostata *and *B. turriformis*. Figure 4 shows the calibrated demographic histories of *G. fasciatus *and *M. herderiana*, and the known lake-level fluctuations and diatom abundance through time inferred from sedimentary cores. Start of the population expansions in both species occurred during a period of relatively high water level, and coincided with a period of high diatom abundance c. 25-50 Kyr BP (thousand years before the present).

## Discussion

In this study we compared phylogeographic patterns and demographic histories of species with similar feeding preferences inhabiting the same geographical area of Lake Baikal. Overall, we found three different phylogeographic patterns in the five species investigated. Both *M. herderiana *and *G. fasciatus *exhibit haplotype networks in which a central haplotype is very abundant and widespread, and to which several less common haplotypes are closely related. *Baicalia carinatocostata *and *B. turriformis *display very different pattern, with the same haplotype never found in more than one locality and lacking a central and most abundant haplotype. *Baicalia carinata *shows an intermediate picture, with some relatively abundant haplotypes being found in different localities and rare haplotypes restricted to single sampling localities.

While phylogeographic patterns often reflect habitat availability and connectivity [8,48] our results suggest that intrinsic biological factors might play an important role in shaping the genetic structure of the species analyzed. The southwestern shore of Lake Baikal, which was sampled for this study, has relatively few sandy areas, with a mostly steep, rocky shoreline (Additional file 4). One would thus expect that species inhabiting mostly sandy bottoms would show high geographic substructuring, while species that prefer rocky habitats would exhibit a pattern indicative of relatively uninterrupted gene flow. Our results, however, are only partially supportive of this hypothesis. Concerning *M. herderiana*, it should be noted that although inhabiting mostly rocky areas, this species has been found in both sandy and silted areas [39]. In this regard, *M. herderiana *resembles the generalist amphipod *G. fasciatus*, which lives in both sandy and rocky substrates [49,50] and is further known to easily invade new habitats and occupy places in ecosystems [42,43,51]. These two species show remarkably similar phylogeographic patterns, with our data suggesting high degree of gene flow throughout the studied geographic range. The inferred patterns for the remaining three species analysed, however, highlight the importance of specific biological characteristics other than preferred habitat type. In fact, *B. carinata *and *B. carinatocostata *both live in sandy bottoms, but show rather different phylogeographic patterns. It was reported that *B. carinata *increases its dispersal by laying eggs on the shells of its conspecifics [40,41], and this could explain the difference between phylogeographic structures. Similarly, the rock-dweller *B. turriformis *displays high degree of geographical substructuring, even though significant geographical barriers between rocky habitats in the sampled shore seem absent. This more sedentary species mostly inhabits the surface of steep rocks and cliffs with individuals hanging on each other [41,44] and is known as a strict specialist in regard to its feeding behaviour and overhanging slopes [44]. This high degree of specialization might reduce the dispersal ability of *B. turriformis *due to the lack of suitable habitats available.

For the reconstruction of the demographic history of populations it is important to identify if a sampling set represents a single population. For *M. herderiana *we sampled most of the range of the shore where this species occurs [52] and found very little genetic differentiation. Likewise, for this species most F_ST _values between localities were non-significant. *Baicalia carinata *and *G. fasciatus *occur along whole shoreline of the lake and previous studies [53,54] involving samples from outside the area of the current study showed that individuals of each of the species form single populations along the southwestern shore. In our analysis, not a single pairwise comparison between localities where *G. fasciatus *was found exhibited significant F_ST _values. For *B. carinata*, significant F_ST _values were found between some localities (notably, between comparison involving localities 11 and 12). Similarly, F_ST _values estimated between localities of *B. carinatocostata *exhibited only few significant results. Conversely, genetic differentiation was higher in *B. turriformis*, despite the smaller sample sizes used in this study, and F_ST _analysis revealed significant genetic differentiation between most localities. These results confirm that samples of *M. herderiana*, *B. carinata*, *B. carinatocostata *and *G. fasciatus *represent populations without strong geographical substructuring, and thus are appropriate for reconstruction of demographic histories. Given the higher genetic differentiation in *B. turriformis*, the reconstruction of demographic histories for this species should be interpreted with caution. Recent results (Peretolchina et al. in preparation) suggest that the co-occurring populations of *B. carinata*, *B. turriformis *and *B. carinatocostata *were not influenced by interspecific geneflow during the time period covered by the current study.

Classic neutrality tests did not detect significant departures from neutrality for any of the datasets. However, the most powerful Ramos-Onsins R2 test [55] detected population growth of *M. herderiana*. For *G. fasciatus*, results of neutrality tests were not significant, but had small p-values (p = 0.08 for R2 and p = 0.09 for Tajima's D test). The structure of the haplotype networks of *M. herderiana *and *G. fasciatus*, with a central abundant haplotype and a number of singleton haplotypes, also suggests population growth for these species.

Our demographic reconstructions suggest that population sizes in *B. turriformis*, *B. carinata *and *B. carinatocostata *were rather stable during their evolutionary histories. There are slight trends towards a decline for *B. turriformis *and *B. carinatocostata *as well as slight trend towards population growth for *B. carinata*. However, these slight trends cannot not be taken as evidence for changes in population size because as they appear, the posterior distributions widen. Conversely, BSPs suggest moderate growth for *G. fasciatus*, and dramatic expansion for *M. herderiana*. Figure 3 (d) allows to compare the duration of demographic histories for all species, and one could see that demographic histories of *M. herderiana *and *G. fasciatus *are short, contrary to demographic histories of *B. turriformis*, *B. carinata *and *B. carinatocostata*. Long demographic histories of *B. carinata*, *B. turriformis *and *B. carinatocostata *do not show response to the climatic fluctuations that are known from the paleo-record of the lake, while shorter demographic histories of *M. herderiana *and *G. fasciatus *exhibit strong to moderate growth. It is thus plausible that *M. herderiana *and *G. fasciatus *are relatively recent colonizers of the southwestern shore of Lake Baikal, while the remaining species analysed represent more ancient inhabitants of this area. Alternatively, *G. fasciatus *and *M. herderiana *populations may have recently undergone strong bottlenecks, with the growth detected reflecting the recent recovery from such bottlenecks, while the remaining species could have maintained relatively constant population sizes throughout their histories. To elucidate this, future work could focus on the analysis of nuclear gene diversity, as autosomal and mitochondrial DNA diversity are expected to show different rates of recovery from bottlenecks [56].

Calibration of demographic histories based on molecular sequences is notoriously difficult, particularly when specific rates of molecular evolution are unavailable [57,58]. Nevertheless, such dating can often provide rough time estimates for important events of a species' evolutionary history. After we calibrated demographic histories for populations of *M. herderiana *and *G. fasciatus *by applying available rates of molecular evolution, we found that the start of expansion of populations of these species coincide, and could be estimated to 25-50 Kyr BP (Figure 4). Urabe et al. [32] inferred lake-level variations from seismic surveying and core sampling of the floor of the lake, which appeared to be correlated to changes of the global climate represented by MIS. However, there is no evidence that the drop of the water level due to climate cooling could separate basins of the lake or result in any kind of geographical separation of the fauna inhabiting the southwestern shore. Diatom abundance, that could directly indicate amount of food items available for both species, is shown in Figure 4 (c). The sedimentary core BDP-93-2 from Buguldeika Saddle [21,23] in concordance with cores st2 and st2-PC-2001 from Akademichesky Ridge [59] demonstrate a strongly pronounced interstadial peak at the time c. 25-60 Kyr BP. This suggests that populations of *M. herderiana *and *G. fasciatus *in the southwestern shore of Lake Baikal started expanding during a warm period of relatively high water level, and when the amount of food available was also rather high. While this would indicate that food availability played an important role in the population growth of these species, it should be mentioned that from c. 24 to c. 14 Kyr BP the amount of diatoms in the lake was very much reduced, however the populations of *M. herderiana *and *G. fasciatus *do not appear to have stopped expanding. Data on sedimentary photosynthetic pigments suggests that, despite the reduced bioproductivity of the lake, green algae, diatoms and dinoflagellates were still present in the lake between 16 and 27 Kyr BP [25]. Therefore, it is possible that during this period the abovementioned species relied on other food items. At any rate, the simultaneous growth detected in *M. herderiana *and *G. fasciatus *suggests that environmental factors promoted the population growth of these species in the southwestern shore of Lake Baikal. High resemblance of demographic histories of *M. herderiana *and *G. fasciatus*, a species known to be of high invasive capability, highlights the strong dispersal potential of *M. herderiana *and its ability to expand its population size when environmental conditions are favorable.

## Conclusions

Demographic histories of populations reflect complex interplay between past environmental changes and ecological properties of species. We investigated how five invertebrate species from the same geographical area and with similar food preferences reacted to the environmental changes known to have happened in the lake. We show that intrinsic ecological specialization plays an important role in the demographic response of the species. In particular, high dispersal abilities and lack of strong habitat preference allowed species to find appropriate habitats and expand their populations in response to favourable environmental conditions.

## Methods

### Sampling, DNA extraction, amplification and sequencing

Gastropods were collected by dredge or dives along southwestern littoral of the lake at depths of 5 to 40 meters. After preliminary sorting of benthic samples, gastropods were fixed in 80% ethanol for 24 hours with subsequent ethanol change to 70% solution and kept until DNA extraction. *G. fasciatus *specimens were collected from the shore, using handle-nets, from the depth of 0 to 1.0 m. Specimens were fixed in 96% ethanol. After incubation at 4ºC for two to three days, 96% ethanol was discharged, and the samples were kept at 4ºC in 70% ethanol. The list of sampling localities is shown in Table 1 (for details see Additional file 1). Relatively few individuals of *B. turriformis *and *B. carinatocosta *are used in this study due to a scarce number of samples collected, which is reflective of the rarity of these species [60].

DNA extraction and PCR amplification were performed according to protocols described in Peretolchina et al. [54] for the gastropods and in Gomanenko et al. [53] for *G. fasciatus*. The CO1 fragment of mitochondrial DNA was amplified using the universal DNA primers of Folmer et al. [61]. Sequencing reactions were performed in the forward direction using the Quick Start Kit (Beckman Coulter Inc.). Sequencing was then carried on in either a 373A DNA Sequencer (Applied Biosystems) or a CEQ 8800 DNA sequencer (Beckman Coulter Inc).

### Phylogeography and reconstruction of demographic histories

The DNA sequences were aligned using ClustalW v. 1.4 [62], and resulting alignment was translated to check for the presence of stop codons.

Haplotype networks were constructed using the program TCS v. 1.2.1 [63]. The threshold value of the statistical parsimony algorithm, defining the maximal number of mutational connections between pairs of haplotypes within the same network, was set to 0.95 [64].

For each species, we estimated F_ST _values between pairs of localities in Arlequin v. 3.5 [65]. We estimated F_ST _values using haplotype frequencies, using a distance matrix between haplotypes based on the Kimura's two-parameter model [66] and using a distance matrix between haplotypes based on Tamura-Nei distance [67]. Significance of F_ST _values was estimated using 10 000 permutations, and resulting p-values corrected for multiple testing using the False Discovery Rate procedure of [68].

We used DNA SP v. 5.10.00 [69] to produce mismatch distributions for each species as well as to perform the following tests of neutrality: Tajima's D test [70], Fu's Fs statistics [71] and *R*2 test [55]. In order to distinguish between population growth and selection, we used H statistics [72].

Bayesian skyline plots were constructed using BEAST v. 1.5.1 [73,74]. Substitution model for each dataset was chosen using jModeltest v. 0.1 [75,76] based on the Akaike information criterion [77]. For *B. carinata *and *B. carinatocostata *jModeltest selected the Hasegawa-Kishino-Yano (HKY) model [78] with a proportion of invariable sites (+I) and a gamma distributed rate heterogeneity among the remaining sites (+G). For the remaining three species the best fitting model was HKY. For *G. fasciatus*, we could not obtain values of ESS (Effective Sample Size) exceeding the recommended value of 200 using HKY model proposed by jModeltest, so we applied the GTR substitution model [79]. In order to check if this change of substitution model for *G. fasciatus *affects the recovered demographic history we compared BSPs for both substitution models. BEAST analysis was performed assuming selected substitution models but parameters were estimated from data. We ran chains of 150 million steps for *M. herderiana*, 20 million steps for *B. carinata *and *G. fasciatus*, and 10 million steps for *B. carinatocostata *and *B. turriformis *to obtain in each run ESS values > 200. For each species we performed at least 2 individual runs and compared the results to check for convergence. Data from two independent runs for each species was combined using Log Combiner v1.5.1 [73,74] in order to observe resulting BSPs.

To convert the time scale of demographic histories from substitutions per site into years, we used earlier suggested divergence rate of 1.83%/Myr (million years) for gastropods [80]. Since there is no calibration of molecular clock available for amphipods, for *G. fasciatus *we used average from the reported rates (1.3-1.9%/Myr) of arthropods [81]. Once absolute time scales were obtained for the species' demographic histories, we matched these histories to paleoclimatic events estimated through radiocarbon calibrations of sedimentary cores [21,32].

## Authors' contributions

VF carried out molecular genetic studies on *M. herderiana*, performed the analysis and drafted the manuscript. BN participated in the analysis and helped to draft the manuscript. TP and JP carried out molecular genetic studies on Baicalia spp. and *G. fasciatus*. DS coordinated the design of the study and has been involved in drafting of the manuscript. All authors read and approved the manuscript.

## Supplementary Material

Additional file 1 **Detailed description of each individual used in the study**. Description includes isolate identification, taxonomic status, locality and year of capture, collectors and accession numbers. Click here for file

Additional file 2 **Tables of pairwise F_ST _values between localities for each studied species, with the p-values given in parentheses**. Significant values before correction for multiple testing are marked with asterisk. Significant values after correction for multiple testing are shown in bold. *Loc *is locality number (see Figure 1), N is number of samples. Click here for file

Additional file 3 **BSP reconstructions for *G. fasciatus *using different substitution models**. Comparison of demographic reconstructions using GTR and HKY substitution models. Thick solid lines are median estimates, and thick dashed lines are mean estimates, shades show 95% highest posterior density limits. Click here for file

Additional file 4 **Maps of underwater landscapes of the study area**. Types of bottom substrates at different depths of the lake. The maps were redrawn from Karabanov EB, Sideleva VG, Izhboldina LA, Mel'nik NG, Zubin AA, Zubina LV, Smirnov NV, Parfenova VV, Fedorova LA, Gorbunova LA, Kulishenko YuL. (1990) Underwater Landscapes of Baikal. Novosibirsk: Nauka Publ.,184 pp. (In Russian). Click here for file
