# Supplementary material for: When environmental changes do not cause geographic separation of fauna: differential responses of Baikalian invertebrates
Source: BMC Evol Biol. 2010 Oct 23;10:320. doi: 10.1186/1471-2148-10-320 (PMC2993716; doi:10.1186/1471-2148-10-320)
Supplement: Additional file 2 — Tables of pairwise FST values between localities for each studied species, with the p-values given in parentheses. Significant values before correction for multiple testing are marked with asterisk. Significant values after correction for multiple testing are shown in bold. Loc is locality number (see Figure 1), N is number of samples. [file 1471-2148-10-320-S2.PDF]

Additional file 2 – Tables of pairwise F_{ST} values between localities for each studied species, with the p-values given in parenthesis.

B. carinata

N	*Loc*	2	3	4	7	9	10	11	12
14	2	-							
3	3	0.21(0.202)	-						
5	4	0.27(0.088)	-0.21(1.000)	-					
2	7	0.16(0.358)	0.57(0.208)	0.38(0.047)*	-				
3	9	0.24(0.111)	0.50(0.100)	0.32(0.018)*	0.68(0.104)	-			
2	10	0.22(0.231)	0.85(0.107)	0.71(0.045)*	1.00(0.336)	0.90(0.101)	-		
7	11	**0.29(0.001)**	0.26(0.038)*	**0.30(0.004)**	0.29(0.025)*	0.20(0.067)	0.33(0.015)*	-	
21	12	0.40(0.018)*	**0.77(0.008)**	**0.53(0.001)**	1.00(0.029)*	**0.89(0.007)**	1.00(0.028)*	**0.34(0.001)**	-

B. carinatocostata

N	*Loc*	2	4	5	9	13
2	2	-				
6	4	0.95(0.036)*	-			
4	5	0.90(0.065)	**0.82(0.004)**	-		
5	9	0.54(0.052)	**0.37(0.002)**	0.45(0.018)*	-	
2	13	1.00(0.332)	0.68(0.078)	0.82(0.068)	-0.02(0.318)	-

B. turriformis

N	*Loc*	4	5	6	7	8
3	4	-				
5	5	**1.00(0.019)**	-			
10	6	**0.89(0.003)**	**0.74(0.002)**	-		
3	7	1.00(0.100)	**1.00(0.018)**	**0.94(0.004)**	-	
4	8	**1.00(0.026)**	**1.00(0.007)**	**0.94(0.002)**	**1.00(0.028)**	-

G. fasciatus

N	*Loc*	1	5	6	7	10	12	14
7	1	-						
17	5	0.41(0.016)*	-					
7	6	-0.17(0.999)	0.41(0.014)*	-				
4	7	0.12(0.177)	0.36(0.030)*	0.12(0.172)	-			
3	10	0.12(0.245)	0.34(0.122)	0.12(0.243)	-0.04(0.880)	-		
2	12	0.75(0.027)*	0.61(0.006)*	0.75(0.024)*	0.55(0.072)	0.61(0.099)	-	
3	14	0.12(0.249)	0.34(0.134)	0.12(0.234)	-0.04(0.887)	0.00(0.588)	0.61(0.103)	-

M. herderiana

N	*Loc*	2	4	5	6	7	10
5	2	-					
4	4	-0.08(0.655)	-				
16	5	0.26(0.049)*	**0.37(0.005)**	-			
19	6	0.04(0.279)	0.10(0.120)	-0.01(0.999)	-		
9	7	0.06(0.192)	0.14(0.051)	0.07(0.359)	-0.02(0.889)	-	
25	10	0.27(0.026)*	0.27(0.032)*	**0.38(0.001)**	**0.35(0.000)**	**0.31(0.009)**	-

Significant values before correction for multiple testing are marked with asterisk. Significant values after correction for multiple testing are shown in bold. *Loc* is locality number (see figure 1), N is number of samples.
