# Supplementary figures and images for: When environmental changes do not cause geographic separation of fauna: differential responses of Baikalian invertebrates
Source: BMC Evol Biol. 2010 Oct 23;10:320. doi: 10.1186/1471-2148-10-320 (PMC2993716; doi:10.1186/1471-2148-10-320)

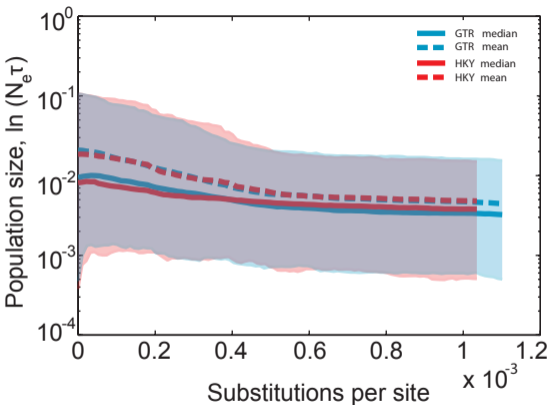

Supplement: Additional file 3 — BSP reconstructions for G. fasciatus using different substitution models. Comparison of demographic reconstructions using GTR and HKY substitution models. Thick solid lines are median estimates, and thick dashed lines are mean estimates, shades show 95% highest posterior density limits. [file 1471-2148-10-320-S3.PDF]
