# Supplementary material for: When environmental changes do not cause geographic separation of fauna: differential responses of Baikalian invertebrates
Source: BMC Evol Biol. 2010 Oct 23;10:320. doi: 10.1186/1471-2148-10-320 (PMC2993716; doi:10.1186/1471-2148-10-320)
Supplement: Additional file 4 — Maps of underwater landscapes of the study area. Types of bottom substrates at different depths of the lake. The maps were redrawn from Karabanov EB, Sideleva VG, Izhboldina LA, Mel'nik NG, Zubin AA, Zubina LV, Smirnov NV, Parfenova VV, Fedorova LA, Gorbunova LA, Kulishenko YuL. (1990) Underwater Landscapes of Baikal. Novosibirsk: Nauka Publ.,184 pp. (In Russian). [file 1471-2148-10-320-S4.PDF]

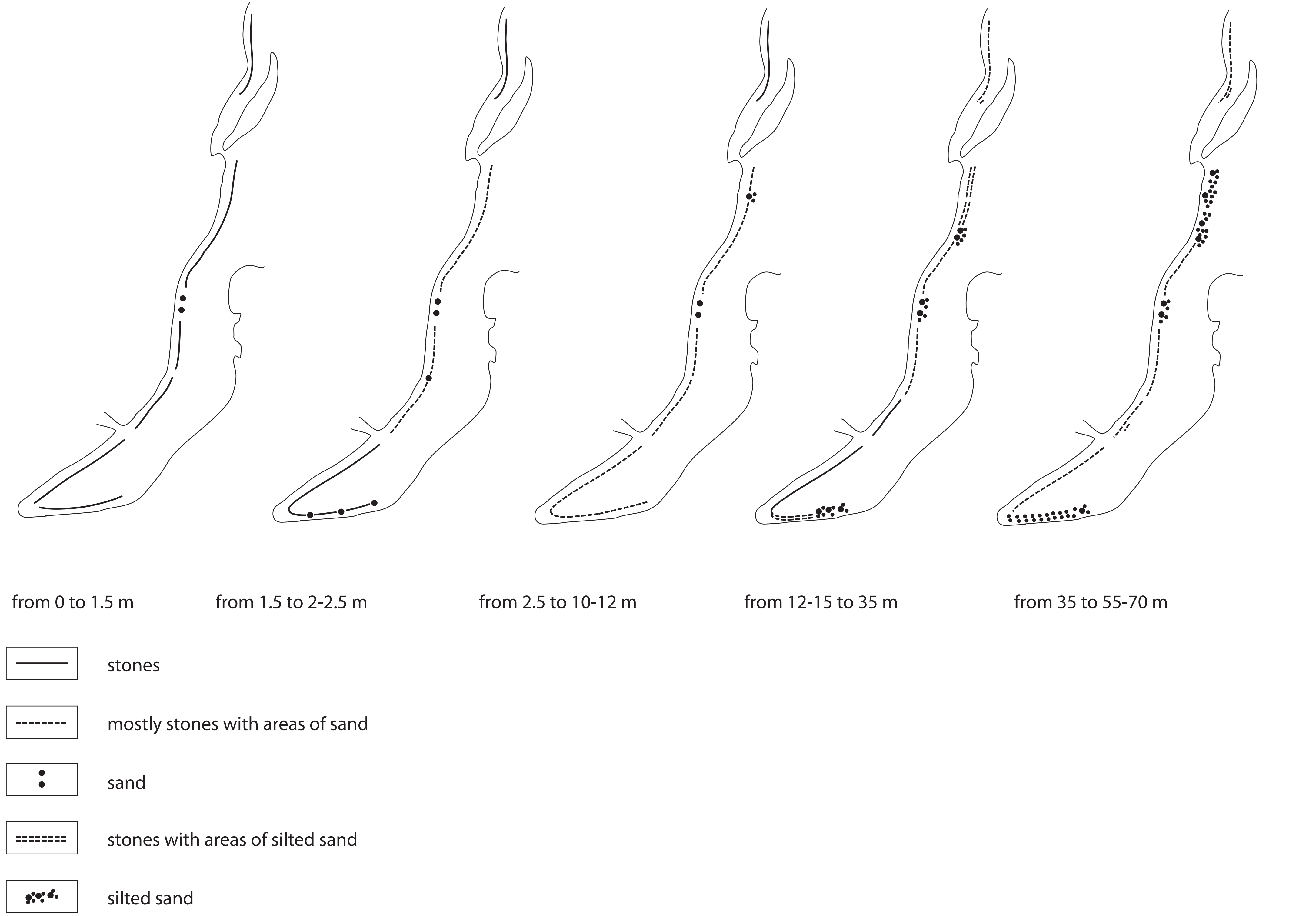
from 0 to 1.5 m
from 1.5 to 2-2.5 m
from 2.5 to 10-12 m
from 12-15 to 35 m
from 35 to 55-70 m
stones
mostly stones with areas of sand
sand
stones with areas of silted sand
silted sand
